# Ultrasound Biomicroscopy Comparison of *Ab Interno* and *Ab Externo* Intraocular Lens Scleral Fixation

**DOI:** 10.1155/2016/9375091

**Published:** 2016-05-18

**Authors:** Lie Horiguchi, Patricia Novita Garcia, Gustavo Ricci Malavazzi, Norma Allemann, Rachel L. R. Gomes

**Affiliations:** ^1^Department of Ophthalmology, Irmandade da Santa Casa de Misericórdia de São Paulo, São Paulo, SP, Brazil; ^2^Department of Ophthalmology, Federal University of São Paulo, São Paulo, SP, Brazil; ^3^Hospital de Olhos Paulista, Rua Abílio Soares 218, 04005-000 São Paulo, SP, Brazil

## Abstract

*Purpose*. To compare* ab interno* and* ab externo* scleral fixation of posterior chamber intraocular lenses (PCIOL) using ultrasound biomicroscopy (UBM).* Methods*. Randomized patients underwent* ab externo* or* ab interno* scleral fixation of a PCIOL. Ultrasound biomicroscopy was performed 3 to 6 months postoperatively, to determine PCIOL centration, IOL distance to the iris at 12, 3, 6, and 9 hours, and haptics placement in relation to the ciliary sulcus.* Results*. Fifteen patients were enrolled in the study. The* ab externo* technique was used in 7 eyes (46.6%) and the* ab interno* in 8 eyes (53.3%). In the* ab externo* technique, 14 haptics were located: 4 (28.57%) in the ciliary sulcus; 2 (14.28%) anterior to the sulcus; and 8 (57.14%) posterior to the sulcus, 6 in the ciliary body and 2 posterior to the ciliary body. In the* ab interno* group, 4 haptics (25.0%) were in the ciliary sulcus, 2 (12.50%) anterior to the sulcus, and 10 (75.0%) posterior to the sulcus, 4 in the ciliary body and 6 posterior to the ciliary body.* Conclusions*.* Ab externo* and* ab interno* scleral fixation techniques presented similar results in haptic placement.* Ab externo* technique presented higher vertical tilt when compared to the* ab interno*.

## 1. Introduction

Capsular bag is the standard of care for posterior chamber intraocular lens (IOL) placement. However, if the capsular support is absent, many techniques can be used to fixate the lens.* Ab interno* and* ab externo* are trans scleral suture techniques described to fixate a posterior chamber intraocular lens (PCIOL) [1–3]. Nevertheless, other techniques to fixate an IOL have been described, such as the no-suture technique that places the IOL haptic inside a scleral tunnel [4–6]. The aim of fixation is to position the haptics in the ciliary sulcus; however, these procedures are performed without direct visualization of the path of the needle.

Direct view techniques, guided by endoscopic probe, have greater rate of success in lens centration and correct haptic position [7, 8]. This technique is considered the gold standard; however, it requires particular equipment and specific training.

Ultrasound biomicroscopy (UBM) is an effective method to study the anterior segment and haptics placement behind the iris [9]. In this study we compared* ab interno* and* ab externo* techniques to evaluate both haptics position in relation to ciliary sulcus and the IOL distance from iris plane determining vertical and horizontal tilt using UBM.

## 2. Methods

This study was approved by the Santa Casa de São Paulo Ethics Committee. Patients scheduled for IOL implantation were randomly assigned to participate in one of the groups. Scleral fixation was performed after a complete ophthalmic examination. Surgery was scheduled according to patient's intraocular inflammation status. Patients with any sign of active inflammation or best-corrected visual acuity worse than 20/40 during the recruitment timeframe were excluded from this study. In one group, the IOL was fixated using* ab interno* technique and in the other group, it was fixated using* ab externo*. All patients did not present adequate capsule support and were candidates for scleral IOL fixation surgery. Block randomization method was used to allocate patients in the treatment groups. All surgeries were performed by the same surgeon (RLRG).

In the* ab externo* surgical technique, two triangular scleral flaps were created at the 2 and 8 o'clock positions in the right eyes and at 4 and 10 o'clock positions in the left eyes. Next, a 26-gauge needle was used to penetrate the bed of the scleral flap at 2 or 4 o'clock position perpendicular to the sclera and 1.5 mm posterior to the limbus. At the same time, a straight needle attached to a 10-0 polypropylene suture was used to penetrate the bed of the opposite scleral flap perpendicular to the sclera. The suture was threaded into the barrel of a 26-gauge needle. An uninterrupted intersulcus suture was placed, extending across the posterior chamber. The anterior chamber was then opened and filled with 2% methylcellulose solution. The suture was pulled outside the eye, divided, and tied to the haptics of a single-piece (polymethylmethacrylate) IOL. The IOL was then placed in the posterior chamber.

In the* ab interno* technique, 2 scleral flaps were created using the same criteria as in the* ab externo* technique, and 2 straight needles with 10-0 polypropylene sutures were passed through a scleral incision parallel to the undersurface of the iris. Further steps were as in the* ab externo* technique.

Ultrasound biomicroscopy (UBM Vumax II, Sonomed Inc., NY, USA) was performed by the same examiner (PNG) under immersion technique (anesthetic drop instillation prior to the insertion of an immersion cup between the eyelids, filled with 10 mL saline solution) from 3 to 6 months postoperatively to detect the position of both haptics in relation to the ciliary sulcus and the position of the optics in relation to the iris, in different positions: 3, 6, 9, and 12 o'clock hours.

Statistical analysis was performed using Stata 14 software (StataCorp 2015, Stata Statistical Software, Version 23, College Station, Texas, USA). IOL tilt in vertical and horizontal planes was compared using variance analysis. IOL tilt was defined as the difference in millimeters in the position between measurements obtained in the vertical plane (12 and 6 hours) and the horizontal plane (3 and 9 hours). Null represented a centered IOL. Positive values represented an anterior displacement of the IOL portion in relation to the perfect position, and negative values represented a posterior displacement of the IOL portion in relation to the null position. A *p* value less than 0.05 was considered statistically significant.

## 3. Results

A total of nineteen eyes of 19 patients were included in the study. Four patients were excluded for lost follow-up. Surgery was performed in 8 right eyes (OD) and 7 left eyes (OS). The mean age was 63.53 ± 20.8 years.

**Figure 1:**
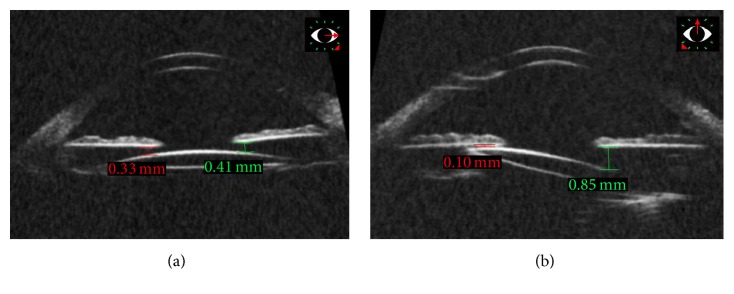
Ultrasound biomicroscopy of the left eye submitted to intraocular lens fixation using* ab externo* technique. (a) Horizontal plane showing good lens positioning; (b) vertical tilt of the IOL, lens placed more posteriorly in the superior meridian compared to the inferior meridian.

**Figure 2:**
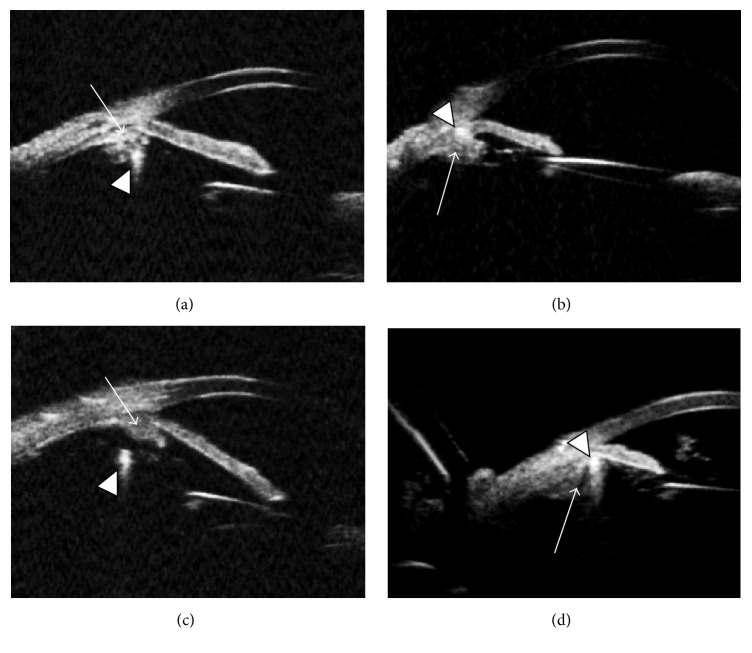
Ultrasound biomicroscopy of eyes submitted to intraocular lens fixation using UBM images. Arrows show the ciliary body, and arrow heads show the haptic. (a)* Ab externo*, haptic placed in the ciliary body; (b)* ab interno*, haptic in the sulcus; (c)* ab interno*, haptic posterior to the ciliary body; (d) haptic in the sulcus.

The* ab externo* technique was used in 7 eyes (57.8 ± 28.4 years, 3 females) and the* ab interno* in 8 eyes (68.5 ± 10.7 years, 5 females). Sixteen eyes were aphakic, 2 had subluxated lens, and 1 had dislocated IOL. Patient demographics and aphakia etiology are listed in Table 1. Figures 1 and 2 demonstrate the UBM images.

In the* ab externo* technique, 14 haptics were located using UBM: 4 (28.57%) in the ciliary sulcus, 2 (14.28%) anterior to the sulcus, and 8 (57.14%) posterior to the sulcus (6 were in the ciliary body and 2 were posterior to the ciliary body). In the* ab interno* group, 16 haptics were located using UBM: 4 (25.0%) in the ciliary sulcus, 2 (12.50%) anterior to the sulcus, and 10 (75.0%) posterior to the sulcus (4 were in the ciliary body and 6 were posterior to the ciliary body). Table 1 summarizes the demographics of the sample and the position of the haptics after the surgical intervention in each group (*ab externo* and* ab interno*) based on information from ultrasound biomicroscopy evaluation.

Tables 2 and 3 compare the findings of intraocular lens position (“tilt”) considering each surgical technique, utilizing ultrasound biomicroscopy.

## 4. Discussion

The aim of scleral fixation techniques is to place the IOL haptics in the sulcus. The results of our study demonstrate that the* ab interno* technique is similar to the* ab externo* to assure the ciliary sulcus placement of the haptics. Four (28.57%) haptics of the* ab externo* technique patients were in the sulcus, compared to 4 haptics (25%) in the* ab interno* group. Using UBM, Kamal et al. reported that 29% of the haptics were in the ciliary sulcus with the* ab interno* technique and 31% with the* ab externo, *with no statistical significance [2].

The results may vary between studies. Pavlin et al. [10] found 38.24% of the haptics in the sulcus, 38.24% anterior to the sulcus, and 23.53% posterior to the sulcus after* ab externo* scleral fixation. Steiner et al. [11] reported 33%, 17%, and 50%, respectively, versus 55%, 27.5%, and 17.5% reported by Sewelam et al. [12]. de Camargo Vianna Filho et al. [13] observed that there was a tendency of the haptics to be placed out of the sulcus in the* ab externo* technique (75%), and this was more evident at one side of the fixation. The current study presented similar results. In the* ab externo* group, 75.0% of the haptics were out of sulcus, and in the* ab interno* group, 71.42% of the haptics were out of sulcus.

Ultrasound biomicroscopy was performed from 3 to 6 months postoperatively. After surgery, all cases had a centered and stable IOL positioned with no IOL-related iris complication. The IOL remained stable during the study follow-up period. In the literature, it is observed that the haptic position after a sutured scleral fixation does not change over time unless suture breakage occurs. The polypropylene suture showed stability for more than 4 years in the long-term evaluation [14, 15]. Price et al. observed late postoperative dislocation of PCIOL, 7 to 14 years after fixation. More studies will be important to determine the long-term results [16].

The variability of the results and haptic placement may be related to the surgeon personal technique and specific anatomic difficulties of each operated eye. We observed more surgical difficulties in the aphakic patients after phacoemulsification. Probably, the anatomy and the orbital conformation could have compromised the result of the primary surgery and might also have influenced the secondary IOL implantation.

Rau et al. [17] evaluated the PCIOL tilt in reference to the iris plane using UBM. They observed 18.1% tilted PCIOL intentionally implanted in the ciliary sulcus. The evaluation of IOL tilt was different in studies. Vasavada et al. [18] and Loya et al. [19] also studied tilt with the same imaging method. We observed that there was a significant vertical tilt in the* ab externo* group and there was no horizontal tilt in our sample. Hayashi et al. observed significantly greater tilt in the eyes that underwent suture scleral fixation when compared to other techniques [20].

*Ab externo* and* ab interno* scleral fixation techniques presented similar results in haptic placement in the group examined.* Ab externo* technique resulted in a higher vertical IOL tilt when compared to the* ab interno* technique.

## Figures and Tables

**Table 1:** Demographics of the sample and cause and technique used for intraocular lens fixation and postoperative IOL positioning evaluated using ultrasound biomicroscopy.

Age (yrs)	Gender	Cause of inadequate capsular support	IOL fixation surgical technique	Postoperative UBM haptics position in relation to sulcus
77	F	After Phaco aphakia	*Ab interno*	(1) Posterior-pars plana (2) Sulcus
80	M	Decentered IOL	*Ab interno*	(1) Posterior-pars plana (2) Anterior
58	M	Unknown aphakia	*Ab interno*	(1) Posterior-ciliary body (2) Anterior
55	F	After Phaco aphakia	*Ab interno*	(1) Posterior-pars plana (2) Sulcus
82	F	After ICE aphakia	*Ab interno*	(1) Posterior-ciliary body (2) Posterior-ciliary body
73	F	After Phaco aphakia	*Ab interno*	(1) Posterior-pars plana (2) Posterior-pars plana
60	F	After Phaco aphakia	*Ab interno*	(1) Posterior-pars plana (2) Sulcus
63	M	Subluxated cataract	*Ab interno*	(1) Sulcus (2) Posterior-ciliary body
18	M	Subluxated cataract	*Ab externo*	(1) Posterior-ciliary body (2) Anterior
43	F	Subluxated cataract	*Ab externo*	(1) Sulcus (2) Sulcus
69	M	After ECE aphakia	*Ab externo*	(1) Sulcus (2) Posterior-ciliary body
82	F	After Phaco aphakia	*Ab externo*	(1) Sulcus (2) Posterior-pars plana
93	M	After Phaco aphakia	*Ab externo*	(1) Posterior-ciliary body (2) Posterior-ciliary body
72	M	After Phaco aphakia	*Ab externo*	(1) Posterior-ciliary Body (2) Anterior
28	F	Unknown aphakia	*Ab externo*	(1) Posterior-ciliary body (2) Posterior-pars plana

F: female; M: male; IOL: intraocular lens; ECE: extracapsular extraction; Phaco: phacoemulsification; ICE: intracapsular extraction; (1): haptic 1; (2): haptic 2.

**Table 2:** Intraocular lens position in relation to the iris (IOL tilt) in the horizontal plane (3 and 9 h), considering UBM findings.

	*N*	Mean (mm)	SD	95% confidence interval	
				Inferior	Superior
*Ab externo*	7	−0.12	0.1603	−0.4356	0.927
*Ab interno*	8	−0.04	0.1138	−0.2668	0.1793
Total	15	−0.08	0.0933	−0.2628	0.1028

SD: standard deviation; IOL: intraocular lens.

**Table 3:** Intraocular lens position in relation to the iris (IOL tilt) in the vertical plane (12 and 6 h), considering UBM findings.

	*N*	Mean (mm)	SD	95% confidence interval	
				Inferior	Superior
*Ab externo*	7	−0.36	0.1261	−0.6058	−0.1113
*Ab interno*	8	−0.2	0.1059	−0.4101	0.0051
Total	15	−0.28	0.0813	−0.4347	−0.116

SD: standard deviation; IOL: intraocular lens.
